# An *in vivo* pharmacological study: Variation in tissue-accumulation for the drug probucol as the result of targeted microtechnology and matrix-acrylic acid optimization and stabilization techniques

**DOI:** 10.1371/journal.pone.0214984

**Published:** 2019-04-04

**Authors:** Armin Mooranian, Nassim Zamani, Ryu Takechi, Giuseppe Luna, Momir Mikov, Svetlana Goločorbin-Kon, Magdy Elnashar, Frank Arfuso, Hani Al-Salami

**Affiliations:** 1 Biotechnology and Drug Development Research Laboratory, School of Pharmacy and Biomedical Sciences, Curtin Health Innovation Research Institute, Curtin University, Perth, Western Australia, Australia; 2 School of Public Health, Curtin Health Innovation Research Institute, Curtin University, Perth, Western Australia, Australia; 3 Department of Pharmacology, Toxicology and Clinical Pharmacology, Faculty of Medicine, University of Novi Sad, Novi Sad, Serbia; 4 Department of Pharmacy, University of Novi Sad, Novi Sad, Serbia; 5 Stem Cell and Cancer Biology Laboratory, School of Pharmacy and Biomedical Sciences, Curtin Health Innovation Research Institute, Curtin University, Perth, Western Australia, Australia; Medical University of Vienna, AUSTRIA

## Abstract

Type 2 diabetes (T2D) is characterised by β-cell damage and hyperglycaemia. The lipophilic drug, probucol, has shown significant β-cell protective and potential antidiabetic effects, which were enhanced by hydrophilic bile acid incorporation using taurocholic acid and chenodeoxycholic acid. However, probucol has severe cardiotoxicity and a variable absorption profile, which limit its potential applications in T2D. Accordingly, this study aimed to design multiple formulations to optimise probucol oral delivery in T2D and test their effects on probucol absorption and accumulation in the heart. Adult male mice were given a high fat diet (HFD), and a week later, injected with a single dose of alloxan to accelerate T2D development, and once diabetes confirmed, divided into three groups (six to seven mice each). The groups were gavaged a daily dose of probucol powder, probucol microcapsules, or probucol-bile acid microcapsules for three months, and euthanized; and blood, tissues, and feces collected for blood glucose and probucol concentration analyses. Probucol concentrations in plasma were similar among all the groups. Groups given probucol microcapsules and probucol-bile acid microcapsules showed significant reduction in probucol accumulation in the heart compared with the group given probucol powder (p<0.05). Probucol microencapsulation with or without bile acids reduced its accumulation in heart tissues, without changing plasma concentrations, which may be beneficial in reducing its cardiotoxicity and optimise its potential applications in T2D.

## Introduction

Type-2 Diabetes (T2D) arises from interactions between the genetic background and environmental triggers. The genetic background has been studied but with limited major discoveries due to the complexity of the human genome and its association with environmental triggers such as obesity. Genome-wide association studies provide useful information on the correlation between nucleotide polymorphism with the incidence of T2D using a large sample size [1]. These studies can provide information on genetic predisposition to the development and progression of T2D [2]. Damage of pancreatic β-cells exhibit a certain genetic signature that is part of the process of T2D development and progression. In a study by Kirkpatrick C *et al*; authors showed that there is a negative correlation between genes encoding the potassium channel in β-cells and the patients’ body mass index [1]. In another study by Wali J *et al*; the authors reviewed the role of β-cell apoptosis in the development and progression of T2D and concluded that cell damage occurs in two main ways, cellular stress (intrinsic) or activation of apoptotic inducing receptors (extrinsic) [3]. Environmental factors include inactivity and lipid disturbances. Accordingly, T2D therapy needs to target not only hyperglycaemia but also β-cell inflammation and damage, and lipid disturbances.

In T2D, studies have linked disturbances in lipid metabolism (dyslipidaemia) and high levels of free radicals with β-cell inflammation and damage. Robertson R *et al*; reviewed causes and processes involved in β-cell functions in T2D, and concluded that the significant decline in functions, despite optimal drug management, is associated with hyperglycaemia and dyslipidaemia. The authors stated that dyslipidaemia is a main cause for β-cell damage due to oxidative stress and high levels of free radicals [4]. A study by Laybutt D.R *et al*; demonstrated that dyslipidaemia and high levels of particular fatty acids trigger a comprehensive endoplasmic reticulum stress response in β-cells via activation of mRNA levels for transcription factors that bring about cell inflammation, malfunction, and death. The authors concluded that β-cell damage due to dyslipidaemia is time-dependent and endoplasmic reticulum stress in T2D is one of the primary contributing factors to β-cell apoptosis [5]. Accordingly, one way to minimise β-cell damage in T2D is by using a drug with lipid-lowering properties, which normalises dyslipidaemia, eliminates free radicals and oxidative stress and protects pancreatic β-cells.

**Fig 1:**
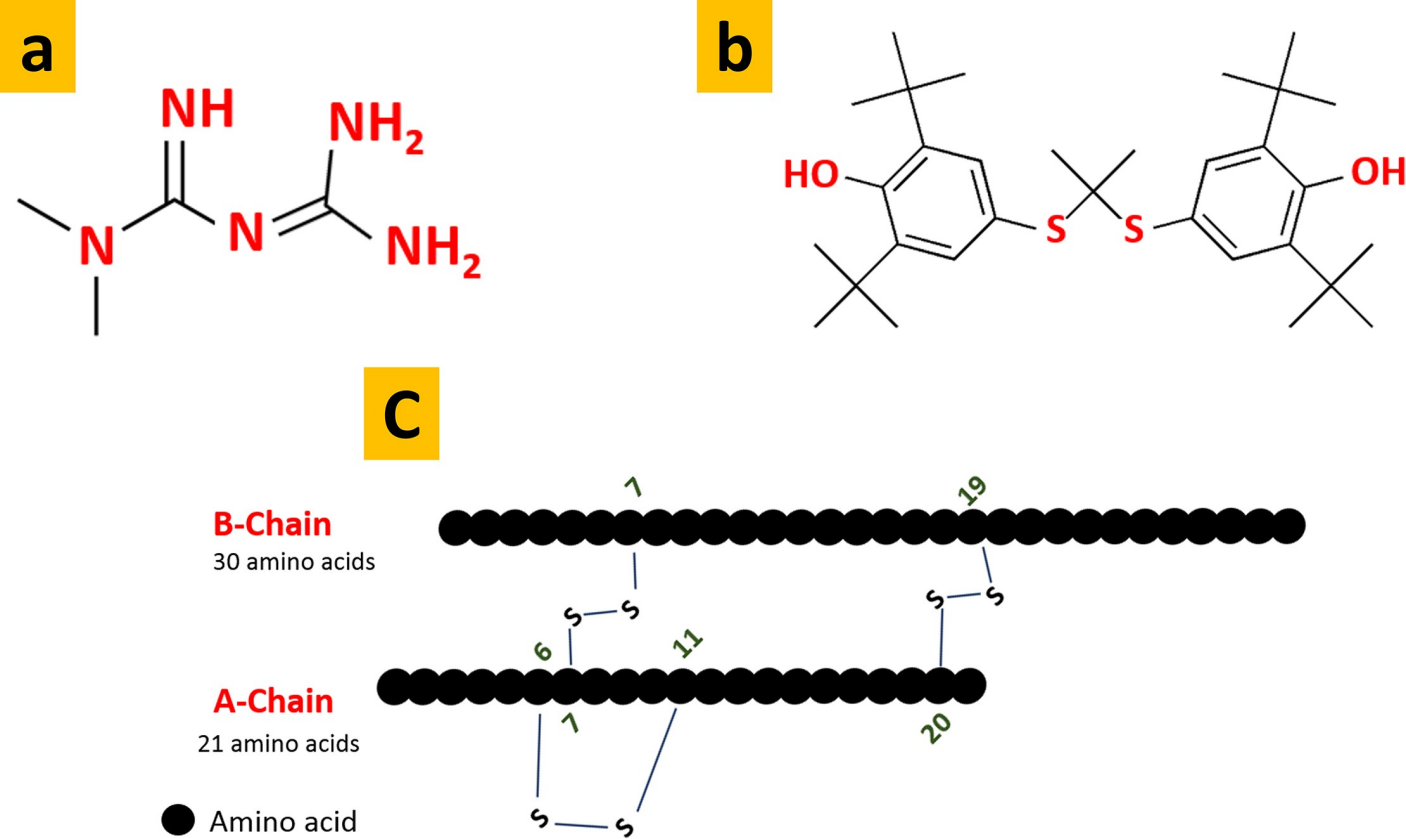
**Chemical structures of metformin (a), probucol (b), and insulin (c)**.

Probucol (Fig 1A) is a drug used for lipid-lowering effects, and has shown significant anti-free radical properties, protects against cellular anti-oxidative stress, and has β-cell protective effects [6–10]. Antidiabetic applications of free probucol have been compared with metformin (Fig 1B) and have shown hypoglycaemic effects at a high dose [11]. Probucol was shown to enhance survival and insulin (Fig 1C) secretion from pancreatic β-cells [10]. However, probucol has been withdrawn from some countries including Australia, owing to its poor absorption profile and high accumulation in tissues and heart, resulting in potentially severe cardiotoxicity (QT interval prolongation). The drug was poor water solubility, has non-linear pharmacokinetics profile with difficult to predict dose-response effects. There are great intra- and interindividual variability in its absorption and therapeutic steady state levels post oral administration owing to its insolubility and varied absorption/permeation in the distal gastrointestinal tract of mammals.

In a recent study in our laboratory, bile acid-based microcapsules containing probucol produced a modest improvement in glycaemic control and reduced the elevated blood glucose levels in insulin-resistant mice, but neither the probucol absorption profile nor cell inflammation were improved [11]. The formulation used in that study failed to achieve satisfactory probucol permeation and absorption after oral administration. Accordingly, in this study, we will use a new probucol formulation composing of a mixture of acrylic acid polymers (Eudragit) incorporated with bile acid-microcapsules. In order to ascertain probucol’s effects on pancreatic β-cells, we will develop a T2D mouse model with significant β-cell damage via using a small dose of the selective β-cell toxin, alloxan, combined with long-term feeding of a high fat diet. The new formulation will be gavaged daily into the mice, long-term, and probucol absorption, tissue and heart accumulation, and antidiabetic effects on glucose levels will be examined.

## Materials and methods

Sodium alginate (SA, 99%), probucol (PB, 98%, C31H48O2S2) and alloxan were all purchased from Sigma-Aldrich (St. Louis, USA). Water soluble gel was supplied by Scharlab S.L. Australia. NM30D (Eudragit polymer) was acquired from Evonik (Vic, Australia). The rodent diets were supplied by Specialty Feeds (Perth, Australia). The water used to dissolve the reagents was HPLC quality purchased from Merck Australia (Sydney, Australia).

Three types of treatments were prepared: probucol powder in an emulsion (10% gel; referred to as probucol powder and this was used as control in the study); alginate-Eudragit microcapsules containing probucol (F1 formulation); and alginate-Eudragit microcapsules containing probucol and the potential antidiabetic bile acid ursodeoxycholic acid (F2 formulation). Probucol microcapsules alone and with ursodeoxycholic acid were produced using a Büchi-based system established in our laboratory, which consisted of a divalent ionic bath (for hardening and forming the microcapsule gel-matrix microstructure) and vibrational frequency guided laminar jet flow established technology, which allowed for precision-guided microdroplet formation via voltage/current mediated microfluidic systems [12–16]. Parameters were set in frequency range of 1100-1500Hz, a flow rate of 2–4 ml/min, a consistent air pressure of 300 mbar, and a nozzle-gelation bath distance of 8cm. The microencapsulation of probucol was confirmed via drug encapsulation efficiency and loaded drug content studies using dissolution assays coupled with quantitative analyses using spectrometry as outlined previously [6–10, 14, 17–19].

### Mice acclimatisation

Adult white Balb/c male mice (purchased from the Animal Resources Centre, Western Australia) were given one-week acclimatisation, with free access to bottled water and food, and were kept at 22°C under a 12-hour dark/light cycle. The experiments were approved by the Animal Ethics Committee at Curtin University and all experiments were performed according to the Australian Code of Practice for the care and use of animals for scientific purposes.

### Type 2 diabetes induction and study protocol

**Fig 2:**
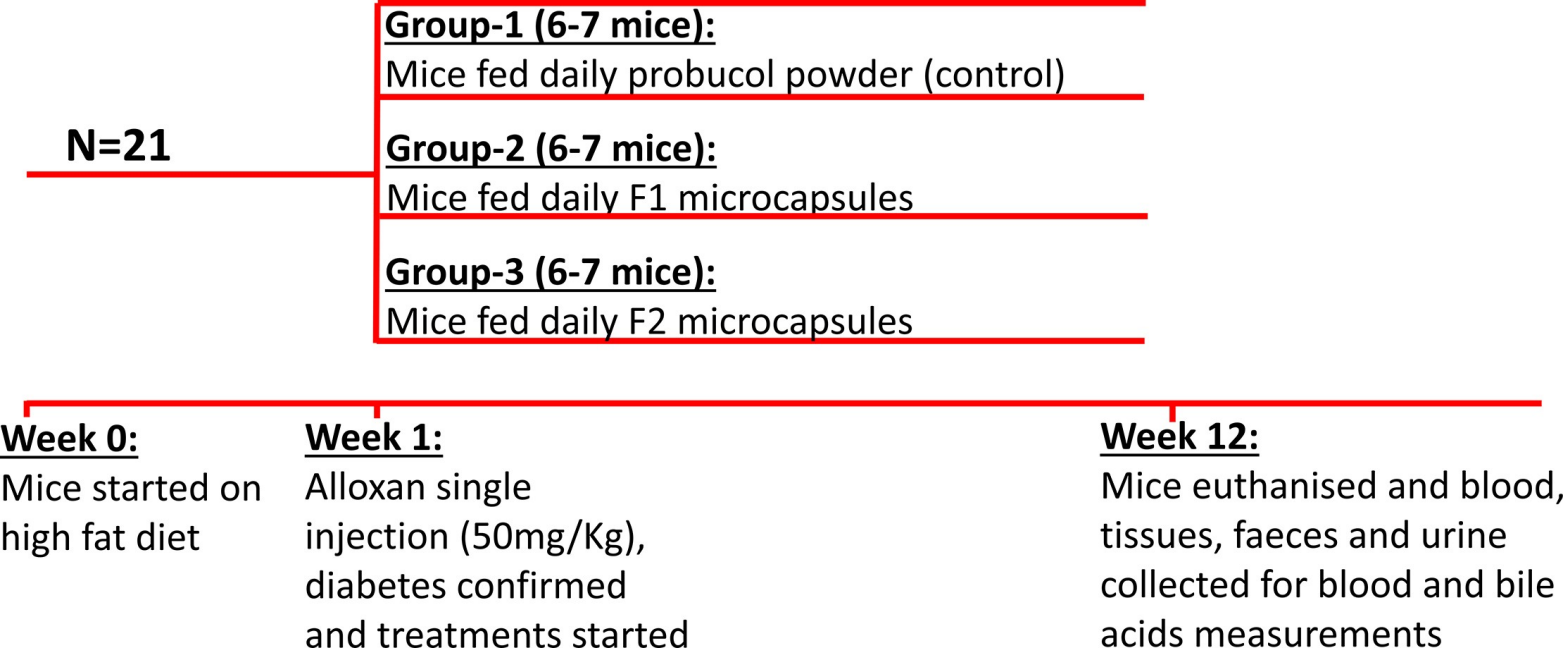
A summary of study design.

Adult white Balb/c male mice (22±3g), six to seven mice per group, were given a high fat diet daily (HFD) *ad libitum*, and a week later, injected with a single dose of alloxan (50 mg/Kg; IP/SC), and divided into three equal groups (seven mice per group). Diabetes was confirmed after two consecutive blood glucose readings of > 13mM taken 24 hours apart. The groups were gavaged a daily dose of probucol powder, probucol microcapsules, or probucol-ursodeoxycholic acid microcapsules (80 mg/Kg) for three months, and euthanized at the end of the study (on day 91) using Isoflurane gas as per approved animal ethics protocols, and then blood, tissues, and faeces collected for blood glucose and probucol analyses (Fig 2).

Blood glucose concentrations (mM) were measured via tail vein venepuncture daily and data analysed using Accu-check glucometers (Roche Laboratories, Basel, Switzerland). Insulin was measured using an ultrasensitive mouse Elisa kit (Mercodia, Switzerland), while the homeostasis model assessment for insulin resistance (HOMA-IR) and β-functions (HOMA-β) was measured mathematically as described elsewhere [20, 21].

### Measurement of inflammatory biomarkers

Concentrations of the inflammatory biomarkers Interleukin (IL)-1β, -12, and -17 were measured using BD Mouse Flex Sets (BD Biosciences, San Jose, California), utilising an Attune Acoustic Focusing Flow Cytometer (Life Technologies, Carlsbad, California, USA), as described elsewhere [22].

### Probucol high-pressure liquid chromatography analysis

High-pressure liquid chromatography (HPLC) was developed and validated for probucol analyses in plasma, stomach, small intestine, large intestine, pancreas, liver, spleen, heart, skeletal muscles, brain, white adipose tissues, kidney, urine, and feces, based on our well-established methods [11]. In brief, standard concentrations of probucol in mobile phase acetonitrile in water were prepared for the range of 0.4 to 1000 μg/ml. An autosampler injection volume of pooled samples used was 10 μL per injection. A 250mmx4.6mm Phenomenex Luna C-18 column (5 μm ID) was used, and the UV detector was set at wavelengths of 241nm. The HPLC used was a Shimadzu Prominence consisting of a Shimadzu DGU-20A5 degasser, LC-20AT liquid chromatographer, SIL-20A autosampler, and SPD-20A UV/Vis detector (Japan). The mobile phase consisted of acetonitrile: water as 96:4% v/v ratio. The flow rate was 1.5mL/min with an autosampler injection volume of 10 μL, and the HPLC setup was an isocratic Shimadzu Prominence HPLC system consisting of a Shimadzu DGU-20A5 degasser, LC-20AT liquid chromatographer, SIL-20A autosampler, and SPD-20A UV/Vis detector (Shimadzu, Japan).

### Statistical analysis

Using GraphPad Prism Version 7.1 (GraphPad, USA), statistical analysis was done using parametric/non-parametric or one-way ANOVA as appropriate. A statistically significant difference was reported when p-value < 0.05. Values are expressed as means ± standard error of the mean.

## Results

**Fig 3:**
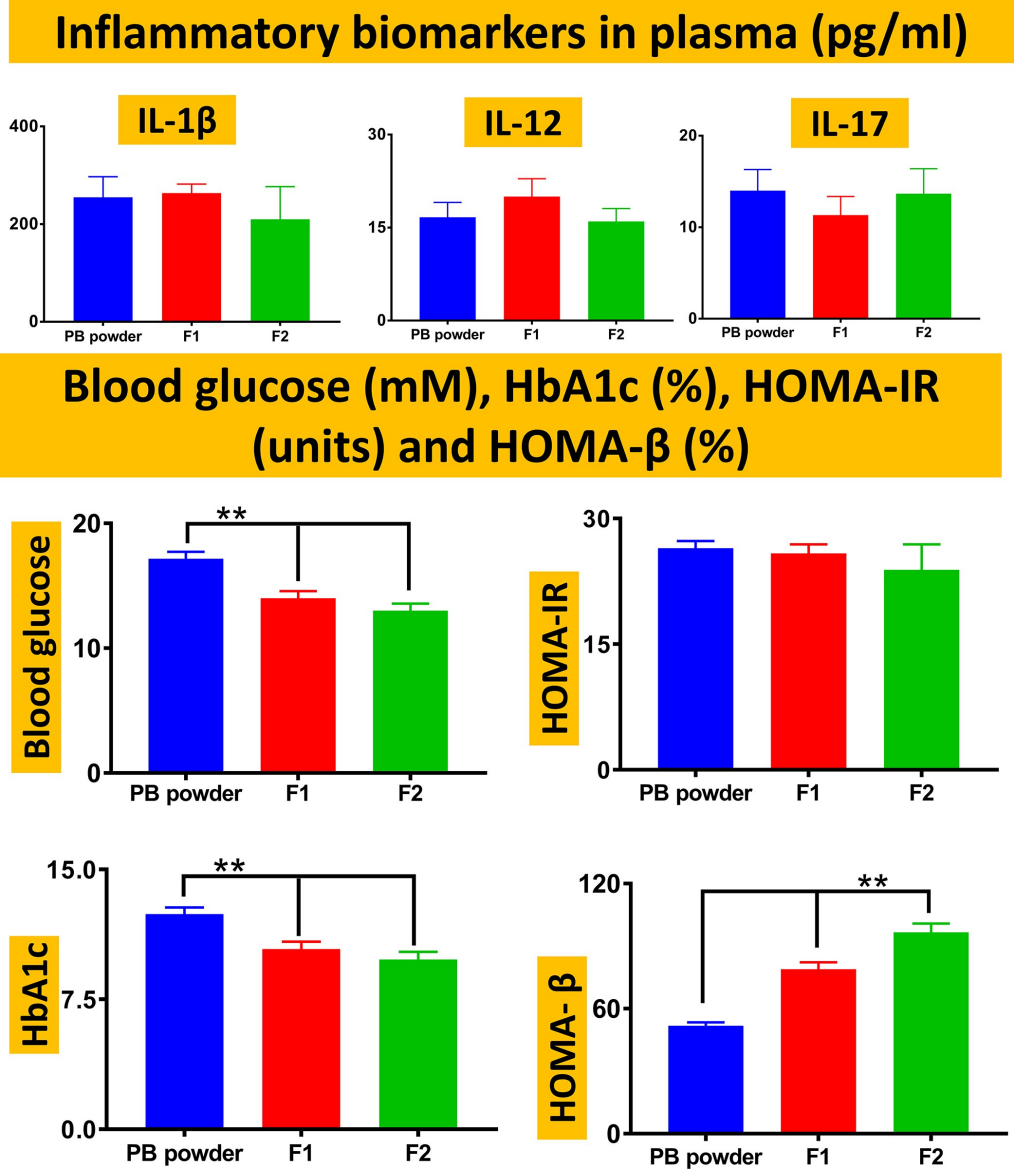
Plasma inflammatory biomarkers, blood glucose, glycated haemoglubin (HbA1c), homeostatic model assessment (HOMA) for insulin resistance (HOMA-IR) and β-functions (HOMA-β). Data are mean ± SEM. ** p < 0.01.

Despite probucol possessing anti-atherosclerotic and weight controlling effects [23], the mice weights remained similar regardless of probucol treatment or formulation used (Table 1). The lack of effects of probucol treatment on mice weight suggests that any potential antidiabetic effects of probucol are likely to be anti-inflammatory or hypoglycaemic (Fig 3).

**Table 1:** Weights of all mice at the beginning, during, and at the end of experiment. Data are average ± SEM, n = 6–7. F1: Alginate-Eudragit microcapsules containing probucol and F2: Alginate-Eudragit microcapsules containing probucol and the potential antidiabetic bile acid ursodeoxycholic acid.

Formulation	Week 0 (weight, g)	Week 6 (weight, g)	Week 12 (weight, g)
**PB powder**	17.5 ± 0.5	21.2 ± 0.7	23.5 ± 1.0
**F1**	18.1 ± 0.4	22.1 ± 0.6	24.6 ± 1.8
**F2**	17.3 ± 0.6	20.5 ± 1.0	23.2 ± 1.3

**Fig 4:**
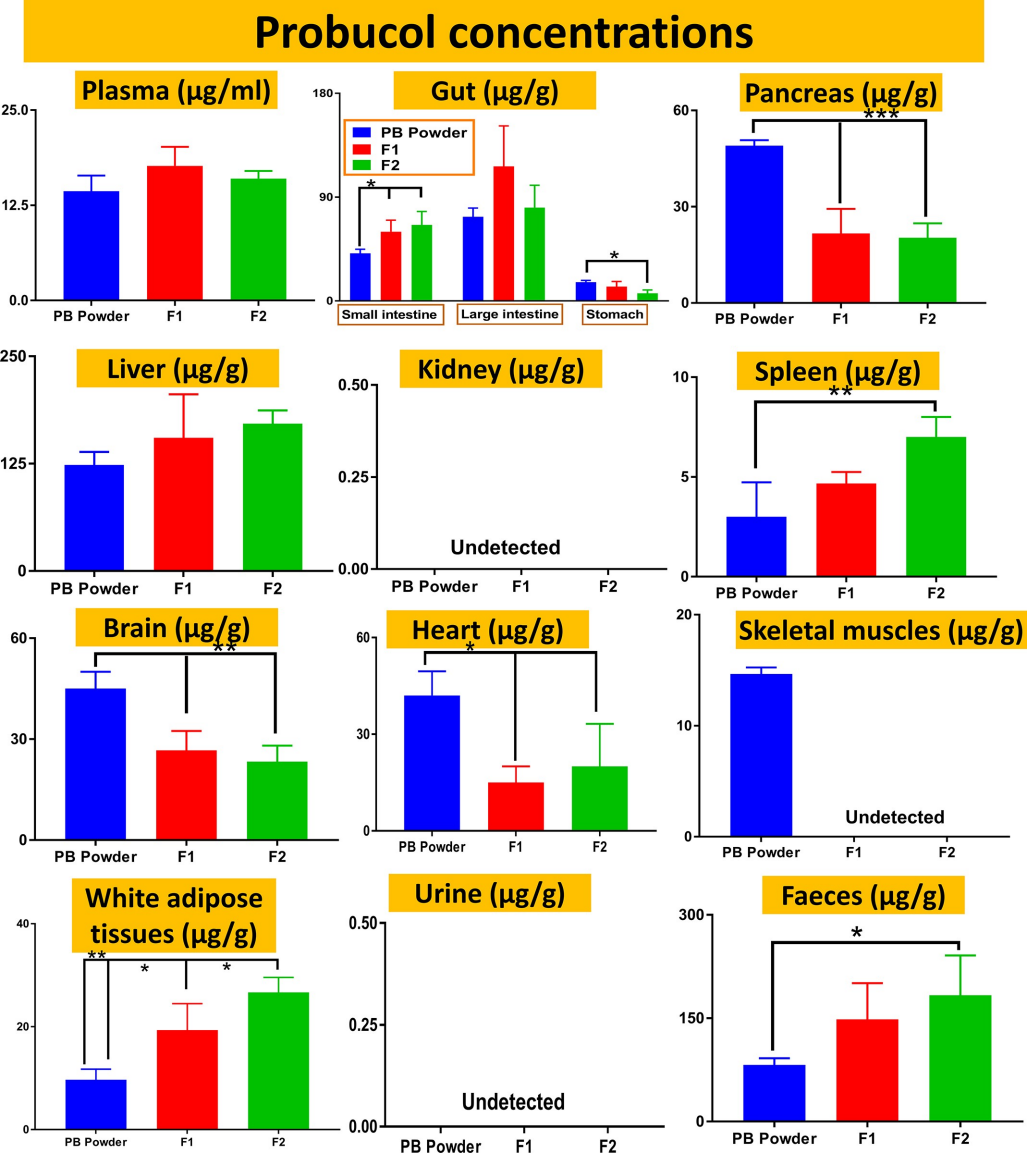
Probucol concentrations in different tissues, urine and feces of treated T2D mice. Data are mean ± SEM. * p < 0.05, ** p < 0.01 and *** p < 0.001.

None of the treamtents exerted signficant anti-inflammatory effects on IL-1β, IL-12 or IL-17, and homeostatic model assessement of insulin-resistance (HOMA-IR) remained similar among all groups, which suggests that none of the treatments reduced inflammation or enhanced tissue sensitivity to insulin (Fig 3). Although all blood glucose and HbA1c levels remained elevated (> 11.1 mM and > 6.5% respectively), both F1 and F2 treatments exerted hypoglycemic effects improving blood glucose, HbA1c, and HOMA-β, which suggests that both F1 and F2 enhanced insulin release and stimulated β-functions. These hypoglycemic effects may be associated with their effects on probucol delivery into blood and tissues (Fig 4).

Plasma concentrations of probucol remained similar regardless of NM30D microencapsulation or ursodeoxycholic acid incorporation, which suggests that systemic absorption was not influenced by F1 or F2 formulation (Fig 4). Stomach epithelial uptake of probucol was reduced mostly by microencapsulation with ursodeoxycholic acid (F2), which suggests that F2 microcapsules showed least release of probucol in the stomach and best targeted release in the lower gut and small intestine, while plasma levels remained comparable raising the possibility of consistent ileal permeation and systemic absorption regardless of gut release. Overall, treatments resulted in increased probucol accumulation in the spleen and white adipose tissues with higher excretion in feces, in particular, F2 microcapsules. This increase was accompanied by a decrease of probucol accumulation in the pancreas, brain, heart, and skeletal muscles, and similar concentrations in the large intestine and liver. This suggests that microencapsulation of probucol, with or without ursodeoxycholic acid, resulted in alteration of its tissue and cellular uptake, and although treatments resulted in improved insulin release and β-functions (Fig 3), that was not directly related to probucol accumulation in the pancreas or skeletal muscles since concentrations of probucol were reduced in these tissues following treatments (Fig 4). Variation in treatments effects on probucol accumulation in different tissues suggests that accumulation is formulation-dependent. the absence of probucol in the urine and kidney suggests that renal metabolism of probucol is not the main route of its excretion, especially when liver uptake was significant regardless of the treatments deployed (Fig 4).

## Discussion

Probucol was initially marketed in the 1970s as a new anti-atherosclerotic drug to treat hypercholesterolemia. Probucol showed significant lipid regulatory effects, but at high concentrations, was associated with prolonged QT intervals and cardiotoxic side effects. Brown KF, *et al*; investigated methods to predicting change in QT interval induced by probucol administration. The authors found that there was prolongation of the QT interval, which was directly associated with increased plasma concentrations following probucol uptake, and these findings linked probucol oral absorption kinetics with its cardiotoxic side effects [24]. Studies in our laboratory have shown optimisation of probucol delivery via microencapsulation technology [6–8, 10, 11]. However, to the best of our knowledge, probucol encapsulation in NM30D-ursodeoxychonlic acid, and investigation of its hypoglycaemic, anti-inflammatory, absorption, and cardiac accumulation profiles in T2D mice, has not been explored. This study complements ongoing work on probucol applications in cardiometabolic disorders [11].

The hypoglycaemic and β-cell supportive effects of probucol (Fig 3) have promising applications in T2D therapy. This is particularly valuable in the absence of weight-modulating effects (Table 1), which is a current health concern when using some antidiabetic drugs [25]. Our results (Fig 4) suggest that probucol’s beneficial antidiabetic effects (Table 1 and Fig 3) were not directly associated with its gut uptake or systemic absorption (Fig 4), but possibly due to its enhanced cellular uptake in adipose tissues, which is a main site for glucose metabolism. It is thus possible that F1 and F2 formulations exerted similar absorption profiles from the gastrointestinal tract (Fig 4) but were capable of enhanced pharmacological activity due to tissue-specific biological effects. Assimacopoulos-Jeannet F, *et al*; have investigated the effects of lipid regulation and glycaemic control on insulin-resistance in adipose and liver tissues. The authors found that in white adipose tissues, there was increased insulin secretion and lipogenic enzymes, which resulted in higher expression of GLUT4 protein and enhanced glycaemic control. The positive glycaemic controlling effects were specific for white adipose tissues, and suggest strong glucose regulatory effects of these tissues, *in vivo* [26]. Other studies have shown that stimulation of glucose uptake in adipose tissues *in vivo* is nitric oxide dependent. Roy D, *et al*; investigated if *in vivo* inhibition of nitric oxide synthase influences insulin-mediated glucose disposal in peripheral tissues. The authors found that levels of nitric oxide underpin insulin stimulation of glucose uptake in adipose tissues and skeletal muscles *in vivo*, which exemplifies the role of adipose tissues in regulating glycaemic control *in vivo* [27]. However, the significant variation of probucol accumulation in tissues such as skeletal muscles, when administered in different formulations, remain unclear and an interesting research question to investigate in future studies.

## Conclusions

Overall, the findings demonstrate potential effects of our microencapsulated probucol in glycaemic control and T2D therapy. Microencapsulated probucol had similar absorption and systemic concentration profiles, but enhanced pharmacological activity. Further studies are needed to ascertain the clinical efficacy of the microcapsules in the management of diabetes mellitus.
